# Network pharmacology and machine learning reveal kit as a target of roburic acid in anaplastic thyroid carcinoma: Preliminary in vitro validation

**DOI:** 10.1371/journal.pone.0359087

**Published:** 2026-10-15

**Authors:** Foxing Tan, Haolin Du, Longcheng Deng, Yaping Shen, Yan Yang, Kun Mei, Zilu Chen, Yan Huang

**Affiliations:** Nanjing Hospital of Chinese Medicine Affiliated to Nanjing University of Chinese Medicine, Jiangsu, Nanjing, China; Universidad El Bosque, COLOMBIA

## Abstract

This study aims to systematically explore the potential molecular mechanism of Roburic Acid (RBA) in the treatment of Anaplastic Thyroid Carcinoma (ATC) through the approaches of network pharmacology, machine learning and in vitro experiments. Public databases were used to retrieve the potential targets of RBA and disease-related targets of ATC. Intersection analysis was performed to identify the common targets, followed by the construction of a Protein-Protein Interaction (PPI) network for these common targets. Enrichment analyses, including GO functional and KEGG pathway analyses, were carried out for the targets. Core targets were further prioritized using SVM, LASSO and random forest machine learning algorithms. Validation of the binding affinity and stability of RBA with the core target was performed using molecular docking and dynamics simulations. Finally, in vitro experiments—including cell viability, scratch assay, apoptosis detection, and Western blot—were conducted using the ATC cell line KHM-5M to validate the therapeutic effect and underlying mechanism of RBA. A total of 401 RBA-associated targets and 2151 ATC-related targets were identified; their intersection yielded 173 shared targets. GO enrichment analysis indicated that these targets were mainly involved in responses to peptide hormones and nuclear receptor activity, among other biological functions. KEGG pathway analysis showed significant enrichment in cancer-related pathways, including the PI3K-Akt and MAPK signaling pathways. Using SVM, LASSO regression, and random forest models, KIT was consistently identified as the single core target. Molecular docking and molecular dynamics simulations demonstrated strong binding between RBA and c-KIT, characterized by low binding energy and the formation of a stable protein-ligand complex. In vitro, RBA significantly reduced KHM-5M cell viability and migration; the inhibitory effect on migration became pronounced at 24 h and was comparable to that of the positive control DOX. Furthermore, RBA promoted apoptosis and dose-dependently suppressed c-KIT phosphorylation. This study integrated network pharmacology, machine learning, and molecular simulations to identify KIT as a high-confidence core target of RBA in ATC. Preliminary in vitro validation demonstrated that RBA suppresses ATC cell viability and migration, induces apoptosis, and inhibits c-KIT phosphorylation in a dose-dependent manner.

## 1. Introduction

Anaplastic thyroid carcinoma (ATC) stands as the most aggressive subtype of thyroid cancer, constituting 1% of all thyroid cancer cases. Despite its low incidence, it accounts for 19.9% of annual thyroid cancer mortality, and a median overall survival of merely 6.5 months [1,2]. Its clinical manifestations encompass rapid cellular proliferation, early dissemination, and resistance to treatment. Approximately 40–50% of patients harbor BRAF^V600E^ mutations, 24% present with RAS mutations, and it is frequently accompanied by advanced-stage mutations such as those in TP53 and TERT, thereby establishing a highly heterogeneous tumor microenvironment [3–5]. Approximately half of all ATC patients harbor distant metastases at diagnosis; concurrently, the rate of surgical resection stands at less than 20%. Although radiotherapy can achieve local control of lesions, it fails to address systemic metastasis. Retrospective investigations have indicated that the one-year survival rate for patients treated with either surgery or radiotherapy alone is less than 20% [6]. Novel combinations of targeted therapy and immunotherapy have extended the median survival from 4 months to 14.5 months [7,8]. However, the treatment cost is substantial, and long-term medication is requisite. Consequently, there is an urgent imperative to develop novel treatment strategies.

The acceptance of Traditional Chinese Medicine (TCM) has increased, with it being widely recognized as an alternative or complementary therapy for cancer sufferers [9]. A mounting body of scientific research and clinical evidence substantiates the substantial positive influence of TCM on enhancing the prognosis of cancer patients [9–12]. Roburic Acid (RBA) is a tetracyclic triterpenoid organic compound extracted from the lipid-soluble fraction of *Gentiana macrophylla Pall* [13]. Although reports regarding the pharmacological activities of RBA are relatively scarce, several extant studies indicate that it exhibits diverse biological activities, encompassing anti-inflammatory, immunomodulatory, and antitumor effects [14–19]. Researchers have found that RBA can effectively suppress the proliferation of diverse tumor cells and induce apoptosis [14]. The mechanism underlying its effects could be intimately associated with the regulation of the NF-κB signaling pathway [19]. These research outcomes comprehensively illustrate the multiple merits and potential application significance of RBA in anti-tumor therapy. Consequently, RBA holds the potential to emerge as a promising treatment approach in the comprehensive treatment of ATC and warrants further in-depth research and clinical exploration.

Network pharmacology is an emerging systems-biology paradigm that departs from the traditional “single-target, single-drug” model by constructing multi-layered networks encompassing disease phenotypes, genes, proteins, and drugs to holistically elucidate therapeutic mechanisms [20–22]. Grounded in the principle that complex diseases such as cancer arise from perturbations in interconnected biological networks rather than isolated molecular defects [23], this approach integrates drug target prediction, protein-protein interaction (PPI) network construction, and functional enrichment analyses to map the topological relationships between a compound and its potential targets [24,25]. Specifically, the workflow typically involves: (i) retrieval of compound-associated targets and disease-related genes from multiple public databases; (ii) intersection analysis to identify candidate therapeutic targets; (iii) construction of PPI networks to reveal hub nodes within the target landscape; and (iv) Gene Ontology (GO) and Kyoto Encyclopedia of Genes and Genomes (KEGG) enrichment analyses to infer underlying biological processes and signaling pathways [26–28]. This multi-database, multi-algorithm strategy provides a comprehensive, data-driven framework for predicting drug actions and prioritizing targets for experimental validation [29].

Nevertheless, network pharmacology carries inherent limitations that must be acknowledged [25]. First, target predictions rely primarily on computational algorithms and database annotations, which may yield false-positive or false-negative results due to incomplete database coverage, species-specific differences, and algorithmic biases [29]. The accuracy of predicted targets remains a concern, necessitating experimental confirmation. Second, PPI networks constructed from high-throughput data often capture binary interactions without accounting for dynamic, context-dependent regulatory relationships within the tumor microenvironmen. Third, enrichment analyses provide statistical associations rather than causal mechanistic insights; pathways identified computationally may not translate directly into pharmacologically relevant events in living systems. Finally, this approach alone cannot quantify binding affinities or predict pharmacokinetic properties. To address these constraints, we integrated three complementary strategies: (i) machine learning algorithms (SVM-RFE, LASSO regression, and Random Forest) to cross-validate and prioritize core targets from the network-derived candidates, thereby reducing reliance on any single computational method; (ii) molecular docking and molecular dynamics simulations to assess binding energetics and complex stability at the atomic level; and (iii) in vitro cellular experiments to functionally validate the computational predictions. This multi-layered validation pipeline strengthens the reliability of findings derived from network pharmacology.

The application of network pharmacology is particularly relevant and advantageous in the context of anaplastic thyroid carcinoma. ATC is characterized by profound inter- and intra-tumoral heterogeneity, driven by co-occurring mutations in BRAF, RAS, TP53, TERT, and other oncogenic drivers, resulting in extensive rewiring of signaling networks that enables rapid adaptation to single-agent targeted therapies. Consequently, therapeutic strategies that consider only one molecular target are frequently inadequate, as parallel pathway activation and compensatory feedback loops rapidly circumvent pharmacological inhibition [30]. The network pharmacology approach directly addresses this challenge by interrogating the disease at the systems level, simultaneously evaluating multiple potential targets and pathways through which RBA may exert anti-tumor activity. By constructing a drug-target-pathway-disease network specific to RBA and ATC, this methodology enables the identification of network hub genes and critical signaling nodes that might otherwise be overlooked by reductionist target discovery approaches [21,24]. Furthermore, given the aggressive nature of ATC and the scarcity of effective systemic therapies, network pharmacology offers a rational, time-efficient framework for repurposing natural compounds such as RBA and for generating mechanistic hypotheses that can guide subsequent experimental and clinical investigations [31].

This research employs a comprehensive approach combining network pharmacology, machine learning, molecular simulation, and in-vitro experiments to systematically examine the mechanism of RBA in the treatment of ATC from the perspectives of protein targets, molecular function, and signaling pathways, ultimately identifying c-KIT as a critical therapeutic target for RBA in ATC.

## 2. Materials and methods

### Materials

#### 2.1.1 Cell line.

The human undifferentiated thyroid cancer cell line KHM-5M was obtained from Wuhan Pronosai Life Science Technology Co., Ltd (China).

#### 2.1.2 Reagents.

The Cell Counting Kit-8 (#K1018) was purchased from APExBIO, USA. The TMR (red) Tunel Cell Apoptosis Detection Kit (#G1502) was purchased from Servicebio Biotechnology, China. The RPMI-1640 basal medium (#PM150110) was procured from Procell Biotech, China. Fetal Bovine Serum (#F102) was purchased from Vazyme Biotech, China. RBA (#6812) was purchased from Yuanye Biotech, China. The Omni-Easy™ One-Step UV Imaging PAGE Gel Rapid Preparation Kit (#PG222) was purchased from Epizyme Biotech, China. c-Kit antibody (#T55359), Phospho-c-Kit antibody (#PC12914), and β-Actin Antibody (#P30002) were purchased from Ab-mart Biotech, China.

#### 2.1.3 Instruments.

The inverted fluorescence microscope (Leica DMI3000B, Germany), the upright fluorescence microscope (Olympus BX43), the cell incubator (HERAcell 150i), Bio-Rad Mini Protean Tetra, Bio-Rad Mini Trans-Blot, and Bio-Rad ChemiDoc XRS+ were used.

### Methods

#### 2.1.4 Target prediction.

The canonical SMILES and two-dimensional chemical structure of RBA were retrieved from the PubChem database (https://pubchem.ncbi.nlm.nih.gov/). Potential protein targets of RBA were predicted using three independent web-based target prediction platforms: PharmMapper (https://www.lilab-ecust.cn/pharmmapper/), Super-PRED (https://prediction.charite.de/subpages/target_prediction.php), and SwissTargetPrediction (https://www.swisstargetprediction.ch/). Predicted targets from the three databases were consolidated, and duplicate entries were eliminated. The resulting target proteins were subsequently converted into official gene symbols using the UniProt database (https://www.uniprot.org).

Target genes associated with anaplastic thyroid carcinoma were retrieved from four public databases: GeneCards (https://www.genecards.org/), OMIM (https://www.omim.org/), Open Targets (https://platform.opentargets.org/), and MalaCards (https://www.malacards.org/). For GeneCards, which provides a quantitative relevance scoring system for disease-gene associations, only targets with a Relevance score ≥ 10 were retained to ensure high-confidence associations. For OMIM, Open Targets, and MalaCards, due to the relatively limited number of ATC-related gene entries available in these databases, all retrieved targets were included without additional score-based filtering. Duplicate genes across the four databases were subsequently removed.

To identify the candidate therapeutic targets of RBA in ATC, the R package “venn” was employed to generate a Venn diagram illustrating the intersection between RBA-associated targets and ATC-related targets. The overlapping genes were retained as the core candidate targets for subsequent network construction and enrichment analyses.

#### 2.1.5 PPI network construction.

Intersecting genes were analyzed using the STRING database (https://cn.string-db.org/) to generate a protein-protein interaction (PPI) network. The analysis was restricted to Homo sapiens, with the minimum required interaction confidence score set to 0.900. The results were then imported into Cytoscape_v3.9.1, and the cytoNCA plugin was utilized to analyze node relationships.

#### 2.1.6 GO and KEGG enrichment analysis.

Using the R packages org.Hs.e.g.,db and clusterProfiler, along with the KEGG rest API, GO and KEGG enrichment analyses were performed and visualized to investigate the underlying biological pathways, functions, and signaling pathways.

#### 2.1.7 Identification of core target.

To pinpoint the most critical driver genes from the list of 173 potential targets, we utilized three distinct machine learning algorithms for feature selection: Support Vector Machine-Recursive Feature Elimination (SVM-RFE), Least Absolute Shrinkage and Selection Operator (LASSO) regression and Random Forest (RF). These methods were applied to anaplastic thyroid carcinoma and normal thyroid expression datasets(GSE33630) from GEO database(https://www.ncbi.nlm.nih.gov/geo/) to identify genes that could effectively discriminate between diseased and normal samples. No ethical approval or informed consent was required for the use of this publicly available dataset.

#### 2.1.8 Molecular docking and molecular dynamics simulation.

3D structure of the core target were sourced from the RCSB Protein Data Bank(https://www.rcsb.org/). Molecular docking was conducted using AutoDock Vina, and the free binding energy was calculated to assess ligand-receptor binding affinity. Ligand-receptor interactions were analyzed and visualized using PyMOL Client.

Molecular Dynamics Simulations were conducted using Gromacs 2022.3. Simulations were performed at constant temperature (300 K) and pressure (1 bar) utilizing the CHARMM36 force field, TIP3P water model for the solvent, and appropriate Na⁺ ions for charge neutralization. We performed energy minimization on the system using the steepest descent method, prior to conducting equilibration simulations, included sequential 1,000,000 steps in both the NVT and NPT ensembles. Simulation processing were performed with 50,000,000 steps, achieving a total simulation duration of 100 ns.

#### 2.1.9 Cell culture and determination of drug concentration.

KHM-5M cells, maintained in complete RPMI-1640 medium (10% FBS) at 37°C/5% CO_2_, were seeded into 96-well plates during the logarithmic growth phase. After overnight culture, cells were treated with RBA for 24 h, 48 h and 72 h. The medium was then removed, and cells were rinsed twice with sterile PBS prior to assessing viability with the Cell Counting Kit-8 for the determination of the optimal RBA concentration. A DMSO vehicle control was included at the corresponding concentration to exclude solvent-specific toxicity.

#### 2.1.10 Scratch assay.

After KHM-5M cells covered the bottom of the 6-well plate, used a sterile pipette tip to scratch vertically across the center of the cell monolayer. Following scratching, the plates were gently rinsed twice with sterile PBS and images were captured under a microscope. Then the cells were treated with 20μM RBA and low concentration serum (2% FBS), while the control group was cultured with the same low concentration serum alone. Images of the scratch gaps were captured at the same positions at 12 h and 24 h post-scratching.

#### 2.1.11 Apoptosis detection.

Cells were divided into different concentration treated groups. Following 24 hours of treatment, apoptosis was assessed using the Apoptosis Detection Kit (G1502). Fluorescence microscopy was used to visualize and record the images, and multiple random fields of view were selected per group. Image J software was employed for overlay analysis of TUNEL-positive signals and DAPI-stained nuclear signals.

#### 2.1.12 Western blot assay.

Cells treated with different concentrations of RBA were lysed using RIPA lysis buffer on ice for 20 minutes. After centrifugation at 10,000 rpm at 4°C for 15 minutes, the supernatant was collected, and protein quantification was performed via the BCA method. A 10% separating gel and 5% stacking gel were prepared for sample loading, electrophoresis, and transmembrane transfer. The membranes were blocked with 5% blocking buffer, followed by incubation with primary antibodies against c-KIT (1:2000), phosphorylated (p)-c-KIT (1:2000), and β-actin (1:5000) at 4°C overnight. After washing, the membranes were incubated with secondary antibody (1:5000) at room temperature for 1 hour. Protein bands were visualized using a chemiluminescence imaging system, and band intensities were quantified using ImageJ software to calculate the relative expression levels of target proteins

#### 2.1.13 Statistical methods.

GraphPad Prism 9.5.0 was employed for comprehensive statistical analysis of all experimental data. All data are presented as mean values ± standard deviation (mean ± SD) to accurately reflect the central tendency and variability of the measurements. For comparisons among multiple experimental groups, one-way analysis of variance (ANOVA) was applied as the primary statistical method.

## 3. Results

### Construction of PPI network and screening of core targets for RBA therapy in ATC

The chemical structure of RBA (Fig 1A) was retrieved from the PubChem database. Potential protein targets of RBA were predicted using three independent databases (SuperPred, PharmMapper, and SwissTargetPrediction). The intersection of these predictions yielded 401 putative RBA target genes (S1 Table), as visualized in the Venn diagram (Fig 1B). Separately, ATC-associated genes were collected from four disease databases (MalaCards, GeneCards, OMIM, and Open Targets), resulting in a consensus set of 2151 disease-related genes (S2 Table), shown in the Venn diagram (Fig 1C). Finally, the overlap between the RBA target genes and the ATC-related genes identified 173 common genes (S3 Table), which represent the core candidate targets for RBA in the treatment of ATC (Fig 1D). This set of overlapping genes was subsequently subjected to functional enrichment analysis.

**Fig 1:**
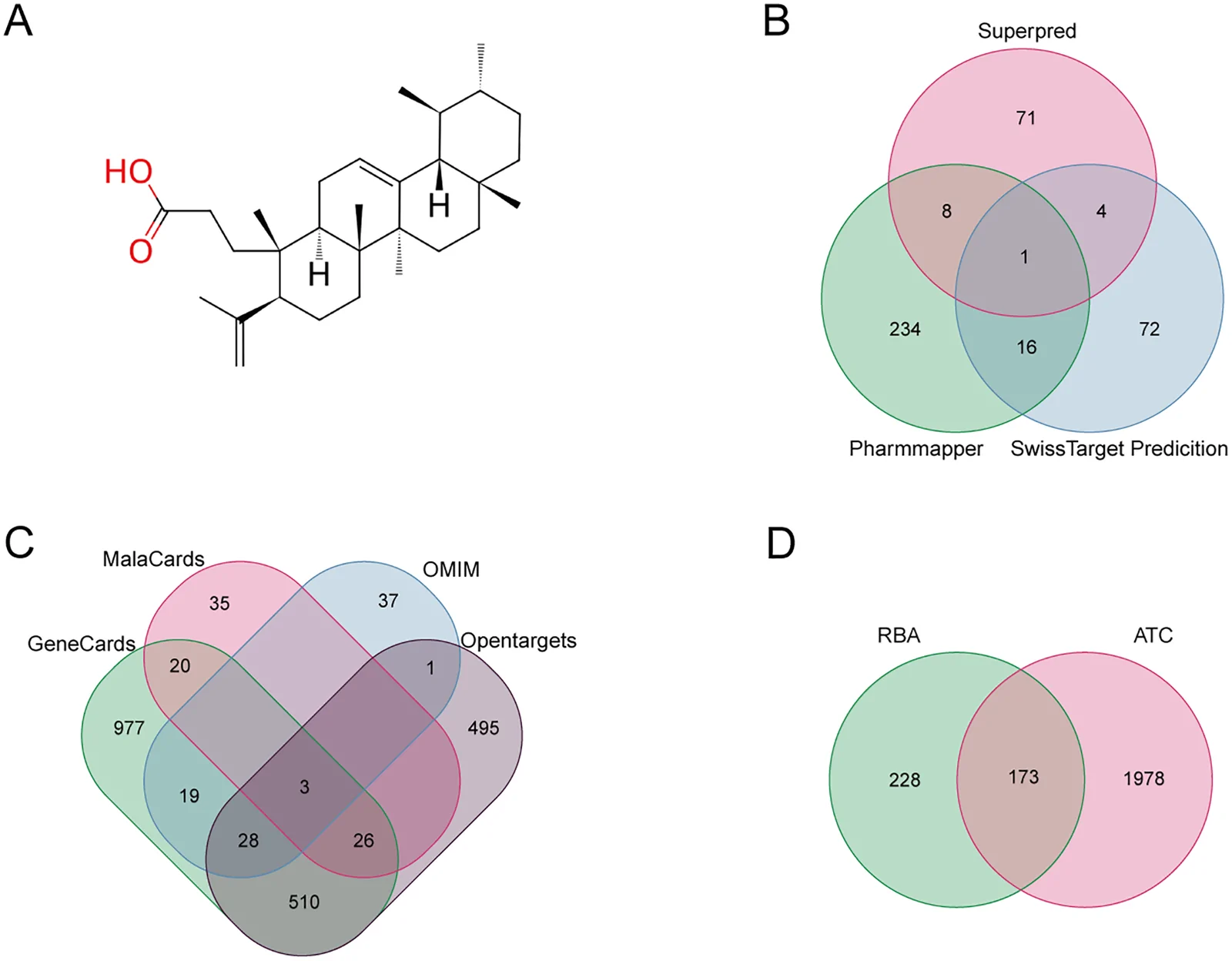
Identification of potential targets of RBA and ATC. (A) Chemical structure of RBA. (B) 401 drug targets from SuperPred, PharmMapper, and SwissTargetPrediction databases. (C) 2151 disease genes from MalaCards, GeneCards, OMIM, and Open Targets databases. (D) 173 overlapping core targets between RBA targets and ATC-related genes.

### PPI network analysis

To explore the functional relationships among these 173 potential targets, we constructed a PPI network using the STRING database (Fig 2A). The network was then visualized in Cytoscape, where node color intensity were scaled proportionally to their degree centrality (Fig 2B).

**Fig 2:**
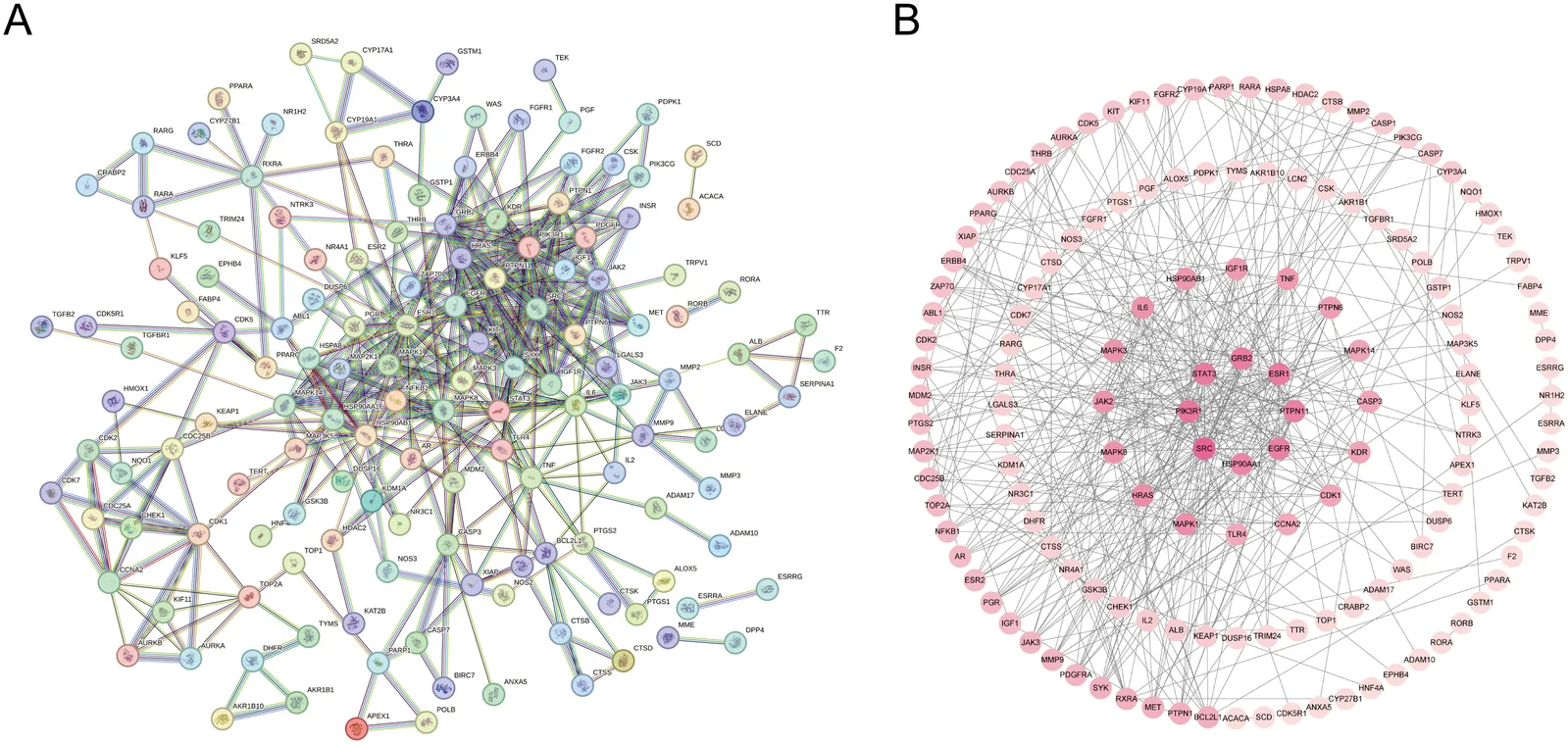
PPI network of RBA-ATC overlapping targets. (A) PPI network generated from STRING database using 173 overlapping genes. (B) Cytoscape visualization of the network.

### GO functional and KEGG pathway enrichment analysis

Gene Ontology (GO) enrichment analysis revealed that in the biological process (BP) category, the target genes were significantly enriched in terms such as response to peptide hormone, cellular response to peptide hormone stimulus, response to molecule of bacterial origin, and the insulin-like growth factor receptor signaling pathway. Regarding cellular components (CC), the genes were primarily localized in structures including membrane raft, membrane microdomain, and ficolin-1-rich granule lumen. Molecular function (MF) analysis indicated that these targets were mainly associated with functions such as nuclear receptor activity, ligand-modulated transcription factor activity, and histone H3Y41 kinase activity (Fig 3A). These findings suggest that RBA may act through modulating hormone response, inflammatory signaling, cell proliferation, diverse kinase activities, receptor-mediated transcriptional regulation, and specific subcellular localizations.

**Fig 3:**
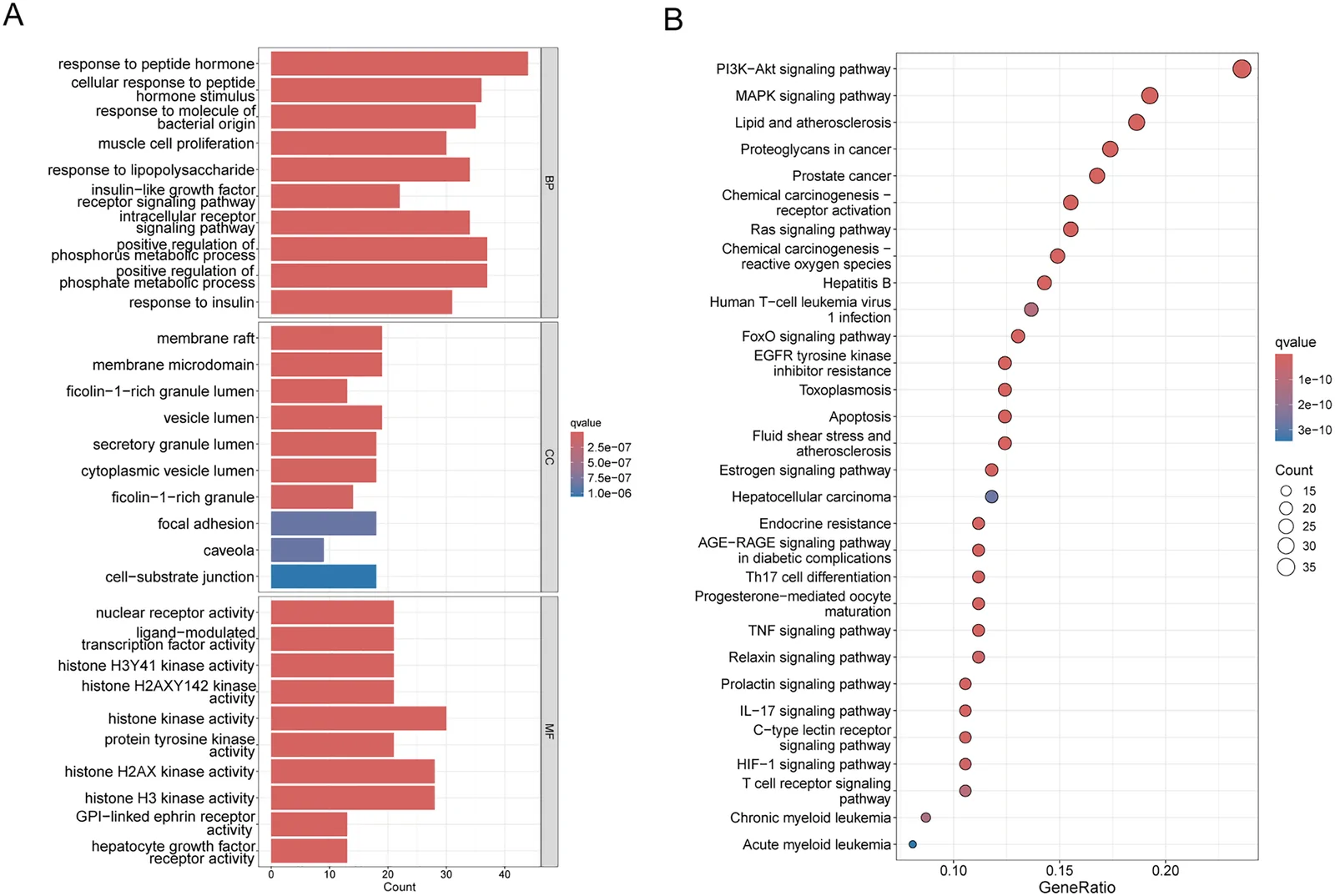
Functional enrichment analysis of 173 core targets. (A) GO enrichment bar plot showing top 10 terms in BP, CC, and MF. (B) KEGG pathway enrichment bubble plot displaying top 30 significantly enriched pathways.

Kyoto Encyclopedia of Genes and Genomes (KEGG) pathway enrichment analysis further identified key signaling pathways associated with the intersecting targets. The genes were significantly enriched in 30 pathways, among which the most prominent included the PI3K-Akt signaling pathway, MAPK signaling pathway, Lipid and atherosclerosis, Proteoglycans in cancer, Prostate cancer, Ras signaling pathway, Chemical carcinogenesis, FoxO signaling pathway, and Apoptosis. Additional enriched pathways of relevance included TNF signaling, AGE-RAGE signaling in diabetic complications, HIF-1 signaling, IL-17 signaling, and EGFR tyrosine kinase inhibitor resistance (Fig 3B).

### Screening of the core target gene using machine learning

To identify key therapeutic targets from the 173 intersecting genes, three distinct machine learning algorithms were applied to gene expression data from the GSE33630 dataset. The SVM model, optimized via 5-fold cross-validation, achieved peak predictive accuracy when the feature set was refined from the initial 29 genes (Fig 4A). Simultaneously, LASSO regression was performed, with the optimal penalty parameter (λ) selected at the point of minimal binomial deviance along the regularization path; this process shrunk the coefficients of most genes to zero, retaining only a minimal set of non-zero predictors (Fig 4B). The RF algorithm was also employed, which ranked all candidate genes by their importance based on the mean decrease in Gini impurity, providing a measure of each gene’s contribution to classification accuracy (Fig 4C). Finally, the results from these three independent analytical approaches were integrated (S4 Table). Their intersection consistently identified a single gene, KIT, as the sole overlapping target selected by all models, highlighting it as a high-confidence core candidate through which RBA may exert its effects in ATC (Fig 4D).

**Fig 4:**
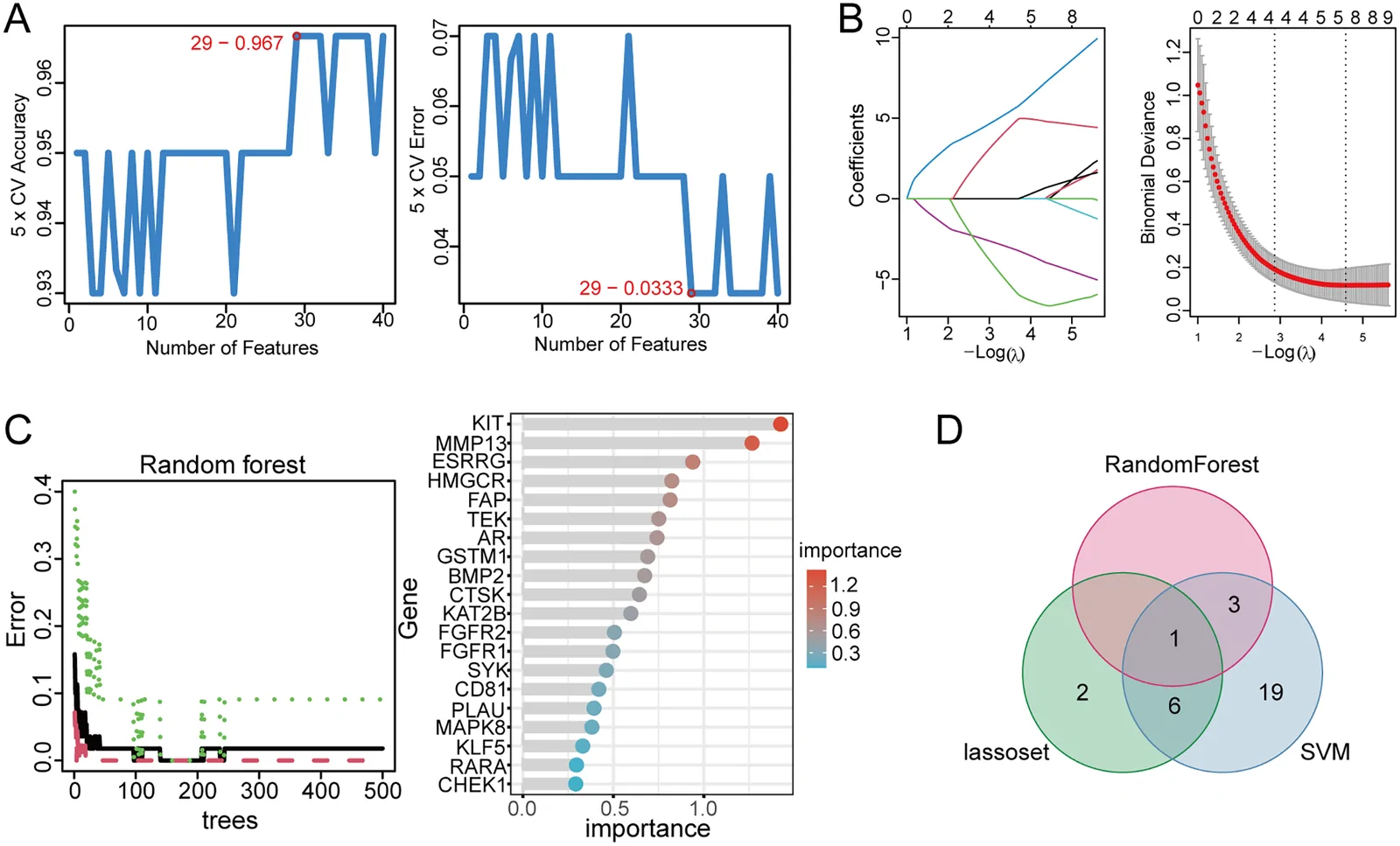
Identification of core targets using machine learning algorithms. (A) SVM feature selection with 5-fold cross-validation. (B) LASSO regression with binomial deviance and coefficient profiles. (C) Random Forest error rate across decision trees and importance ranking of top candidate genes from Random Forest. (D) Venn diagram showing the intersection of genes selected by SVM, LASSO, and Random Forest.

### Molecular docking

To explore the influence of RBA on KIT more comprehensively, in-depth molecular docking investigations were conducted, and the binding energy with RBA was −7.7 kcal/mol. RBA exhibits a strong binding affinity to KIT, forming hydrogen bonds with the active sites of ASP-810, LYS-623 (Fig 5).

**Fig 5:**
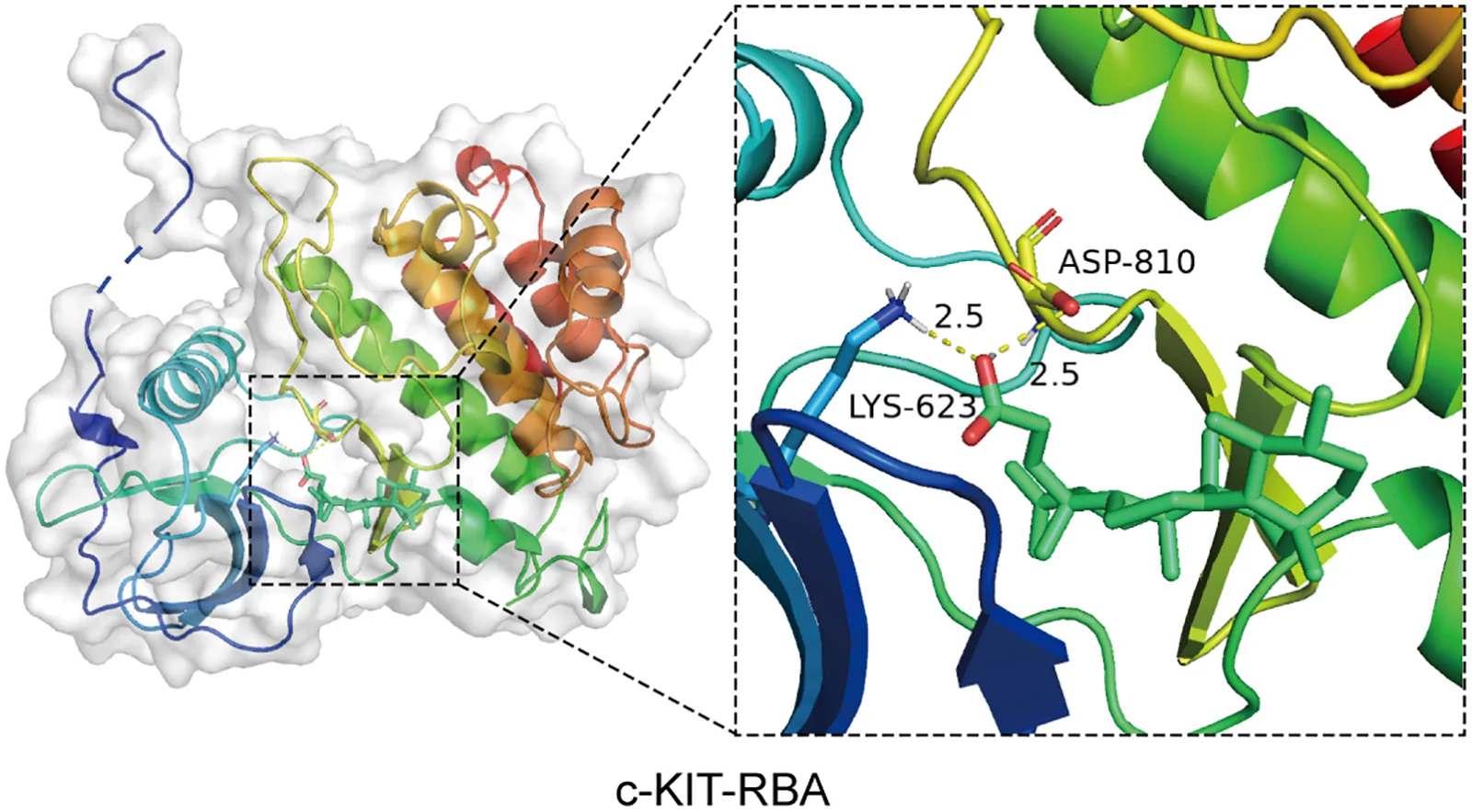
Molecular docking of RBA with c-KIT protein. Key interacting residues (ASP-810, LYS-623) in the binding pocket are highlighted.

### Molecular dynamics simulations of RBA and c-KITProteins

Molecular dynamics simulations evaluated the stability of the c-KIT-RBA complex over a 100 ns trajectory (Fig 6). The root mean square deviation (RMSD) for both the protein backbone and the ligand stabilized rapidly and remained low, indicating a structurally steady complex. Root mean square fluctuation (RMSF) analysis revealed reduced flexibility in residues near the binding site, suggesting ligand-induced stabilization. The interaction was further characterized by consistent hydrogen bond formation and a decreased solvent-accessible surface area (SASA) at the binding pocket, implying stable and specific binding. These results computationally support a stable interaction between RBA and the c-KIT protein, providing a structural dynamics basis for its proposed mechanism.

**Fig 6:**
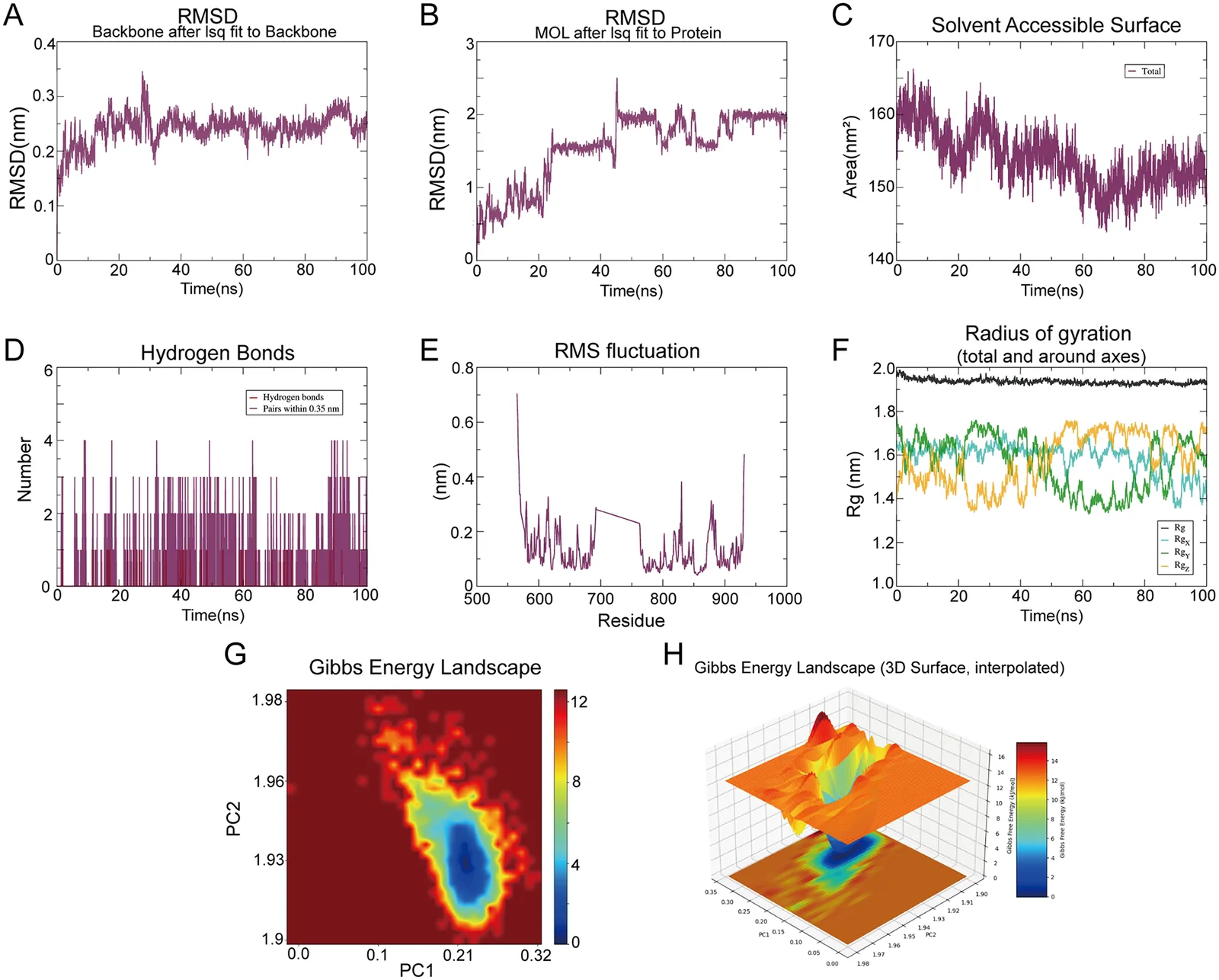
Molecular dynamics analysis confirms the stable binding between c-Kit and RBA (A) RMSD of the c-Kit protein backbone. (B) RMSD of the RBA ligand. (C) SASA of the complex. (D) RMSF of c-Kit residues. (E) Number of hydrogen bonds between c-Kit and RBA. (F) Radius of gyration (Rg) of the complex (including total Rg and Rg around principal axes). (G-H) Gibbs free energy landscape of c-Kit constructed via principal component analysis (G: 2D energy landscape; H: 3D interpolated energy landscape).

### RBA inhibited the proliferative activity of KHM-5M cells

The CCK-8 assay was utilized to validate the effectiveness of RBA at gradient concentrations on KHM-5M cell viability. The results revealed that the IC50 concentrations at 24 h, 48 h, and 72h post-drug administration were 30.11 μM 21.51 μM and 14.39 μM, respectively (Fig 7A-C). And the inhibitory effect of RBA was not attributable to DMSO solvent toxicity (S1 Fig). 20 μM, 40 μM and 60 μM were designated as the low, medium, and high drug concentrations for subsequent experiments.

**Fig 7:**
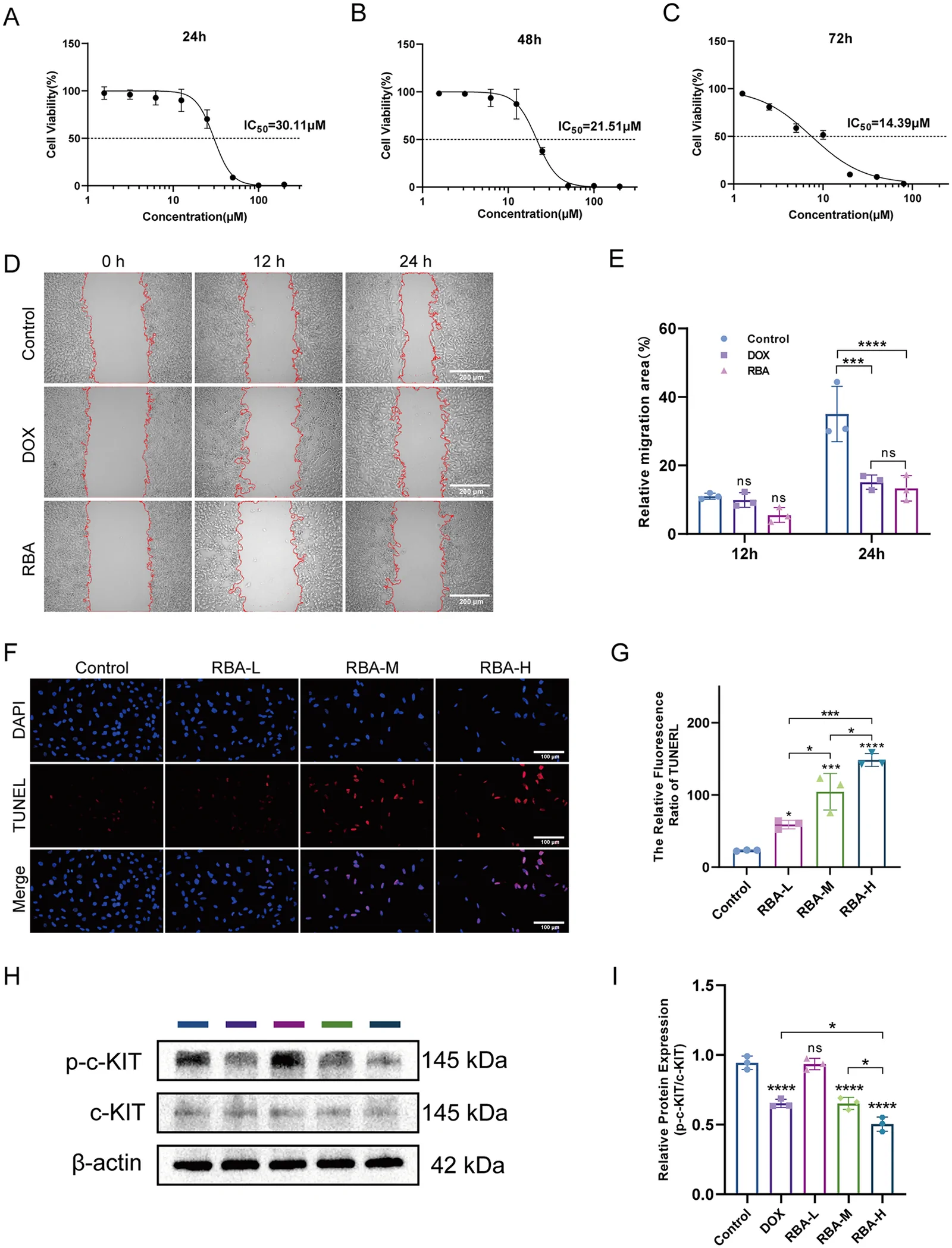
Experimental validation of RBA effects on ATC cells in vitro. (A-C) Cell viability of KHM-5M cells treated with different concentrations of RBA for 24 h 48 h and 72 h detected by CCK-8 assay. (D) Representative images of scratch areas at 0 h, 12 h, and 24 h. (E) Quantitative analysis of the open wound area. (F) Representative images of DAPI (nuclei, blue), TUNEL (apoptotic cells, red), and merged channels. (G) Quantitative analysis of the relative fluorescence ratio of TUNEL signals. (H) Western blot analysis of c-KIT and phosphorylated c-KIT (p-c-KIT) protein expression (I) Quantitative analysis of the relative protein expression levels of p-c-KIT normalized to total c-KIT. Data are presented as mean ± SD. *p < 0.05, **p < 0.01, ***p < 0.001, ****p < 0.0001; ns, not significant.

### RBA reduced the migration rate of KHM-5M cells

The scratch assay results indicated that at the initial stage (0 h), no notable differences were observed in the scratch area among the control, DOX, and RBA groups. At 12 h, cell migration remained comparable across all three groups with no significant differences. However, by 24 h, the control group exhibited significantly accelerated cell migration, with the scratch being substantially closed. In contrast, both RBA and DOX markedly impeded cell migration, with their relative migration areas being significantly lower than that of the control group (p < 0.0001 and p < 0.001, respectively). Notably, no significant difference was detected between the RBA and DOX groups at 24 h (Fig 7D-E). These findings imply that RBA can effectively suppress cell migration, with the inhibitory effect becoming pronounced at 24 h and comparable to that of the positive control DOX.

### RBA induces apoptosis in KHM-5M cells

Based on the TUNEL apoptosis staining results, as the treatment concentration increased, the red TUNEL-positive signal (apoptotic cells) exhibited a significant concentration-dependent elevation, whereas the control group only displayed a small number of spontaneously apoptotic cells. Quantitative analysis further verified that the relative fluorescence ratio of each concentration group was significantly higher than that of the control group (Fig 7F-G). This finding suggests that the treatment can effectively induce apoptosis, and the effect exhibits an obvious concentration-dependent relationship.

### RBA Suppresses c-KIT Phosphorylation in KHM-5M Cells

To further investigate whether RBA inhibits KHM-5M cell migration through modulation of the c-KIT signaling pathway, Western blot analysis was performed to examine the phosphorylation status of c-KIT. Notably, RBA treatment suppressed c-KIT phosphorylation in a dose-dependent manner. Although no significant difference was observed between the RBA-L group and the control group, the RBA-M group and RBA-H group exhibited a significantly reduced p-c-KIT/c-KIT ratio relative to the control group (Fig 7H-I). The results demonstrate that RBA effectively inhibits c-KIT phosphorylation in a concentration-dependent manner, suggesting that the suppressive effect of RBA on KHM-5M cell migration may be mediated, at least in part, through inactivation of the c-KIT signaling pathway.

## 4. Discussion

Anaplastic thyroid carcinoma accounts for 19.9% of all thyroid cancer deaths, making it the leading cause of thyroid cancer mortality [1]. Despite advances in multimodal management incorporating surgery, radiotherapy, and chemotherapy, the majority of patients present with unresectable or metastatic disease at diagnosis, and treatment outcomes remain dismal [32]. Although the advent of targeted therapies, particularly BRAF/MEK inhibitors for BRAF V600E-mutant ATC, has modestly improved survival outcomes, durable responses are rare and acquired resistance remains a significant challenge [33]. These clinical realities underscore the urgent need for novel therapeutic strategies with distinct mechanisms of action.

Natural products derived from traditional Chinese medicine (TCM) have garnered increasing attention as potential sources of novel anti-cancer agents, given their multi-target, multi-pathway modulatory characteristics and generally favorable safety profiles. Roburic acid (RBA), a tetracyclic triterpenoid compound isolated from the roots of Gentiana macrophylla Pall., has been reported to exhibit diverse pharmacological activities, including potent anti-inflammatory effects through NF-κB pathway inhibition, suppression of colorectal cancer cell growth via direct TNF targeting [19], and inhibition of lung cancer metastasis through autophagy induction [17]. Furthermore, RBA has demonstrated remarkable efficacy in ameliorating rheumatoid arthritis symptoms by reprogramming pro-inflammatory macrophage metabolism [15], suggesting its potential for modulating immune and inflammatory responses in the tumor microenvironment. These multi-faceted biological activities position RBA as a promising candidate for ATC treatment, warranting systematic investigation of its molecular targets and mechanisms.

Network pharmacology represents a paradigm shift in drug discovery research, moving beyond the traditional “single drug, single target” approach toward a systems-level understanding of therapeutic interventions [31]. By constructing comprehensive drug-target-pathway-disease networks, this methodology enables the prediction of multi-target drug actions and facilitates the identification of key nodes within complex biological systems. The integration of machine learning algorithms, including Support Vector Machine-Recursive Feature Elimination (SVM-RFE), Least Absolute Shrinkage and Selection Operator (LASSO) regression, and Random Forest (RF), provides robust computational frameworks for feature selection and biomarker identification from high-dimensional transcriptomic data [34]. These complementary computational approaches, when combined with molecular docking and molecular dynamics simulations, offer a powerful strategy for predicting drug-target interactions at the atomic level and assessing the stability of ligand-protein complexes [35]. The present study employed this integrated computational-experimental pipeline to systematically investigate the therapeutic mechanism of RBA against ATC, encompassing target prediction, network analysis, core gene prioritization, binding affinity assessment, and in vitro functional validation.

By integrating gene data from seven databases, we identified 401 potential targets of RBA and 2151 targets associated with ATC, among which 173 were identified as common targets. Subsequent protein-protein interaction (PPI) network analysis and enrichment studies revealed that these targets are significantly involved in biological processes related to peptide hormone response, nuclear receptor activity, and kinase-mediated signal transduction. Notably, KEGG pathway enrichment demonstrated prominent involvement of the PI3K-Akt and MAPK signaling cascades, which are well-established oncogenic pathways in ATC pathogenesis and represent validated therapeutic targets.

The application of three distinct machine learning algorithms—SVM-RFE, LASSO regression, and Random Forest—enabled robust convergence on KIT as the sole core target consistently selected across all models. This convergence substantially strengthens the confidence in KIT as a bona fide therapeutic target of RBA, mitigating the limitations inherent to any single algorithmic approach. Molecular docking analysis revealed favorable binding energy (−7.7 kcal/mol) between RBA and the c-KIT protein, with hydrogen bond formation at critical residues ASP-810 and LYS-623 within the ATP-binding pocket. Furthermore, 100 ns molecular dynamics simulations confirmed the temporal stability of the RBA-c-KIT complex, as evidenced by low RMSD values, sustained hydrogen bonding, and reduced solvent-accessible surface area at the binding interface. These computational findings collectively provide compelling molecular-level evidence for the direct interaction between RBA and c-KIT.

c-Kit (CD117), a transmembrane receptor tyrosine kinase encoded by the KIT proto-oncogene, represents a pivotal regulator of hematopoiesis, melanogenesis, germ cell development, and interstitial cell of Cajal function [36]. Upon binding to its cognate ligand stem cell factor (SCF), c-Kit undergoes homodimerization and autophosphorylation, subsequently activating multiple downstream signaling cascades including the PI3K-Akt, MAPK/ERK, JAK/STAT, and PLC-gamma pathways, which collectively govern cell survival, proliferation, differentiation, adhesion, and migration [36]. The mechanistic basis for KIT-driven ATC progression involves the constitutive or ligand-dependent activation of multiple oncogenic signaling networks. As a class III receptor tyrosine kinase, activated c-Kit recruits adaptor proteins and activates the PI3K-Akt and MAPK/ERK cascades, which promote tumor cell survival by inhibiting pro-apoptotic proteins, enhance proliferation through cyclin-dependent kinase activation, and facilitate migration and invasion via matrix metalloproteinase induction. In addition, the emerging link between KIT and glutamine metabolism introduces a metabolic dimension to its oncogenic function. Glutamine serves as a critical nutrient for cancer cells, providing carbon and nitrogen precursors for biosynthetic pathways, maintaining redox homeostasis through glutathione synthesis, and fueling mitochondrial ATP production via anaplerotic entry into the TCA cycle. By modulating glutamine utilization, KIT may enable ATC cells to meet the metabolic demands of rapid proliferation and adapt to the nutrient-deprived tumor microenvironment.

Based on our computational and experimental findings, we propose a multi-modal mechanism of action for RBA in ATC. Through direct high-affinity binding to the c-KIT ATP-binding pocket, RBA competitively inhibits kinase activity, thereby attenuating downstream PI3K-Akt and MAPK signaling as evidenced by reduced c-KIT phosphorylation in our Western blot analyses. Concurrently, RBA-mediated KIT inhibition may disrupt glutamine metabolic flux, depriving ATC cells of essential biosynthetic intermediates and inducing metabolic stress. Beyond these tumor-intrinsic effects, RBA-induced KIT modulation may potentiate anti-tumor immunity by altering the immune landscape of the tumor microenvironment. The documented correlation between KIT expression and immune checkpoint gene expression (PDCD1 and PDCD1LG2) suggests that KIT inhibition could potentially enhance the efficacy of immunotherapy approaches. The capacity of RBA to simultaneously target tumor cell proliferation, metabolic adaptation, and immune modulation represents a compelling polypharmacological profile that aligns with the complex, multi-factorial nature of ATC.

Our in vitro experimental results provide preliminary validation of this proposed mechanism. RBA treatment significantly reduced KHM-5M cell viability in a dose- and time-dependent manner, with IC50 values of 30.11 μM, 21.51 μM, and 14.39 μM at 24 h, 48 h, and 72 h, respectively. The compound markedly inhibited cell migration in wound healing assays and induced concentration-dependent apoptosis as detected by TUNEL staining. Crucially, Western blot analysis demonstrated that RBA effectively suppresses c-KIT phosphorylation in a dose-dependent manner, with significant reductions observed at medium and high concentrations. These functional data corroborate our computational predictions and establish c-KIT phosphorylation inhibition as a proximal molecular event mediating the anti-ATC effects of RBA.

Several limitations of this study should be acknowledged. First, the target prediction relied on publicly available databases and computational algorithms, which may not capture all potential targets or accurately reflect protein dynamics in the native cellular context. Second, our molecular dynamics simulations, while informative, were conducted over 100 ns; longer simulation times or enhanced sampling methods might reveal additional conformational states and binding interactions. Third, the in vitro experiments were performed using a single ATC cell line (KHM-5M), and validation in additional ATC cell lines and patient-derived models is warranted to generalize our findings. Fourth, the absence of in vivo efficacy data precludes conclusions regarding pharmacokinetic properties, optimal dosing regimens, and anti-tumor activity in an immunocompetent tumor microenvironment. Future studies should address these limitations through comprehensive in vitro characterization across multiple ATC models, in vivo xenograft and patient-derived xenograft studies, and mechanistic investigations employing KIT knockout or overexpression systems to definitively establish c-KIT as the primary target mediating RBA anti-ATC activity.

## 5. Conclusion

In conclusion, this study integrates network pharmacology, machine learning, molecular docking, molecular dynamics simulations, and in vitro experiments to elucidate the mechanism of RBA in ATC. KIT was identified as a high-confidence core target through convergent analysis across three machine learning algorithms. Computational analyses demonstrated stable, high-affinity binding of RBA to c-KIT, and in vitro experiments confirmed that RBA suppresses ATC cell viability and migration, induces apoptosis, and inhibits c-KIT phosphorylation in a dose-dependent manner. These findings establish c-KIT as a plausible primary target mediating the anti-ATC effects of RBA and provide a rationale for further in vivo validation and potential clinical development.

## Supporting information

S1 Table Predicted target genes of Roburic Acid (RBA) from PharmMapper, Super-PRED, and SwissTargetPrediction databases. (XLSX)

S2 Table Anaplastic Thyroid Carcinoma (ATC)-related target genes retrieved from GeneCards, OMIM, Open Targets, and MalaCards databases. (XLSX)

S3 Table Intersection of RBA-associated targets and ATC-related targets (overlapping core candidate targets). (XLSX)

S4 Table Core target genes identified by SVM-RFE, LASSO regression, and Random Forest machine learning algorithms. (XLSX)

S1 Fig Cytotoxicity assessment of DMSO vehicle control on KHM-5M cell viability. (TIF)

S1 raw images Original and uncropped raw blot images. (PDF)

## Abbreviations

RBA: roburic acid
ATC: anaplastic thyroid carcinoma
PPI: protein-protein interaction
TCM: traditional Chinese medicine
GO: gene ontology
KEGG: Kyoto encyclopedia of genes and genomes
BP: biological processes
CC: cellular components
MF: molecular functions
SVM-RFE: Support Vector Machine-Recursive Feature Elimination
LASSO: Least Absolute Shrinkage and Selection Operator
RF: Random Forest
