# Supplementary material for: Network pharmacology and machine learning reveal kit as a target of roburic acid in anaplastic thyroid carcinoma: Preliminary in vitro validation
Source: PLoS One. 2026 Oct 15;21(10):e0359087. doi: 10.1371/journal.pone.0359087 (PMC13671499; doi:10.1371/journal.pone.0359087)

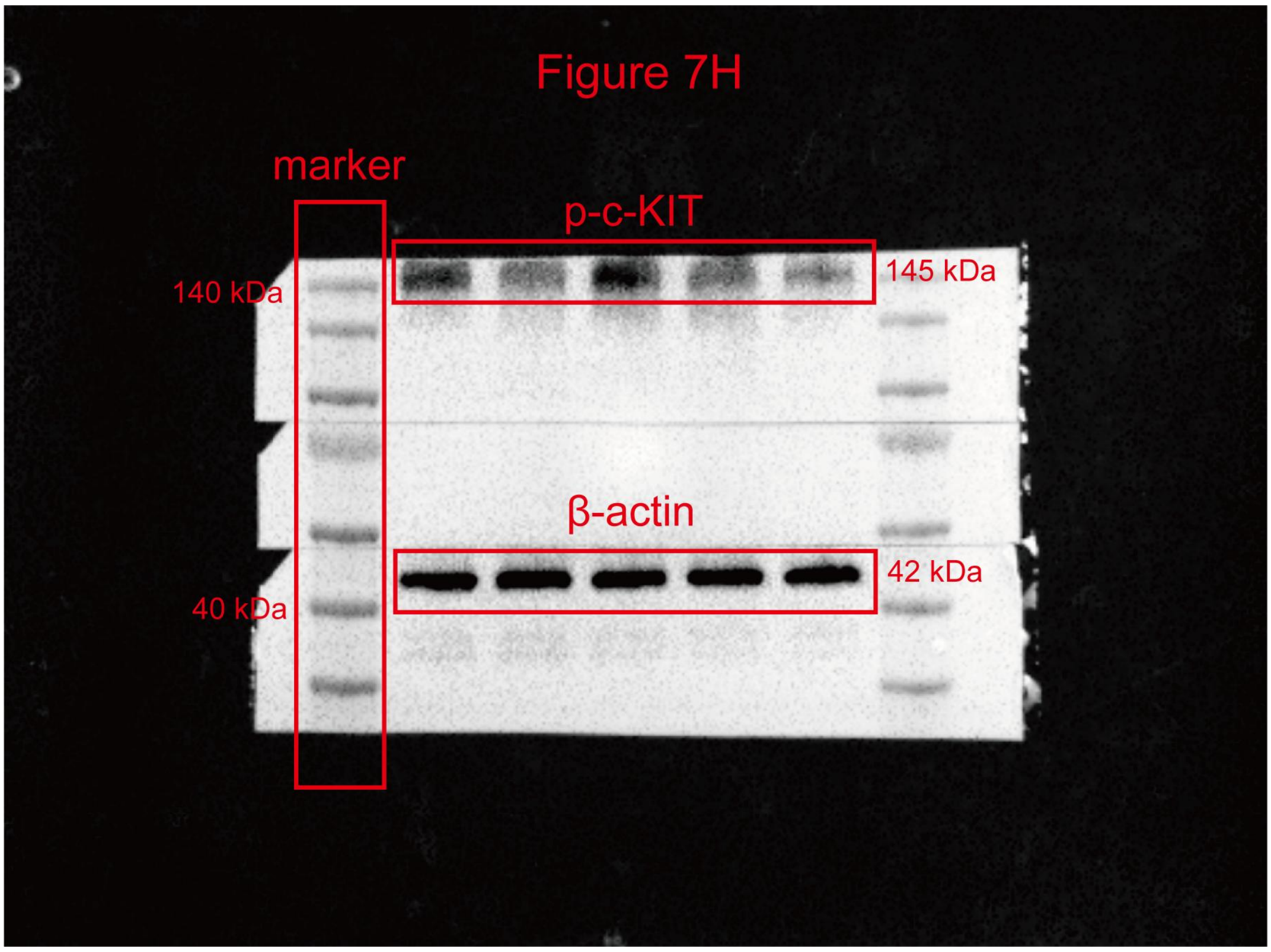
Figure 7H
marker
p-c-KIT
140 kDa
145 kDa
β-actin
42 kDa
40 kDa

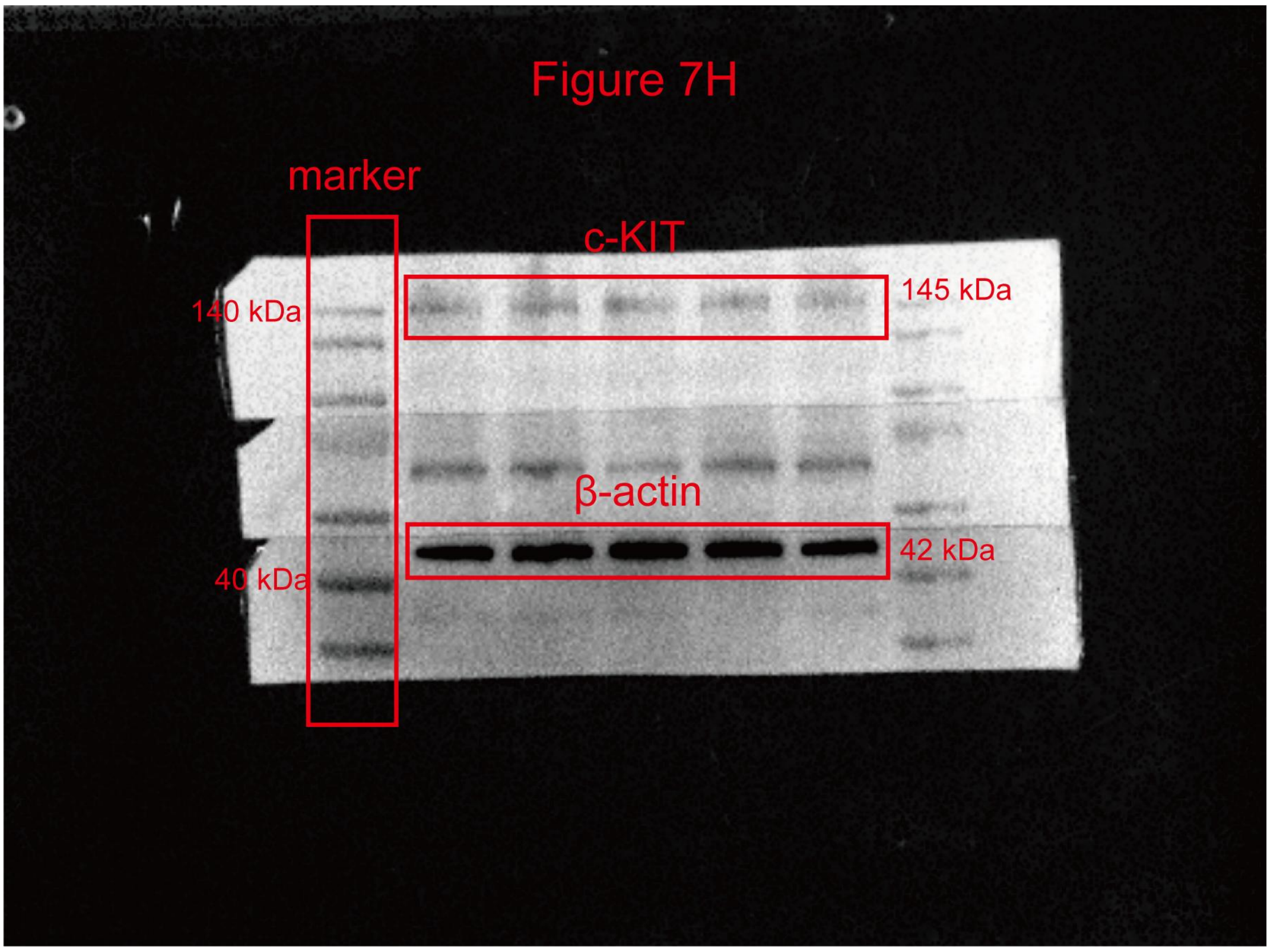
Figure 7H
marker
c-KIT
140 kDa
145 kDa
β-actin
42 kDa
40 kDa

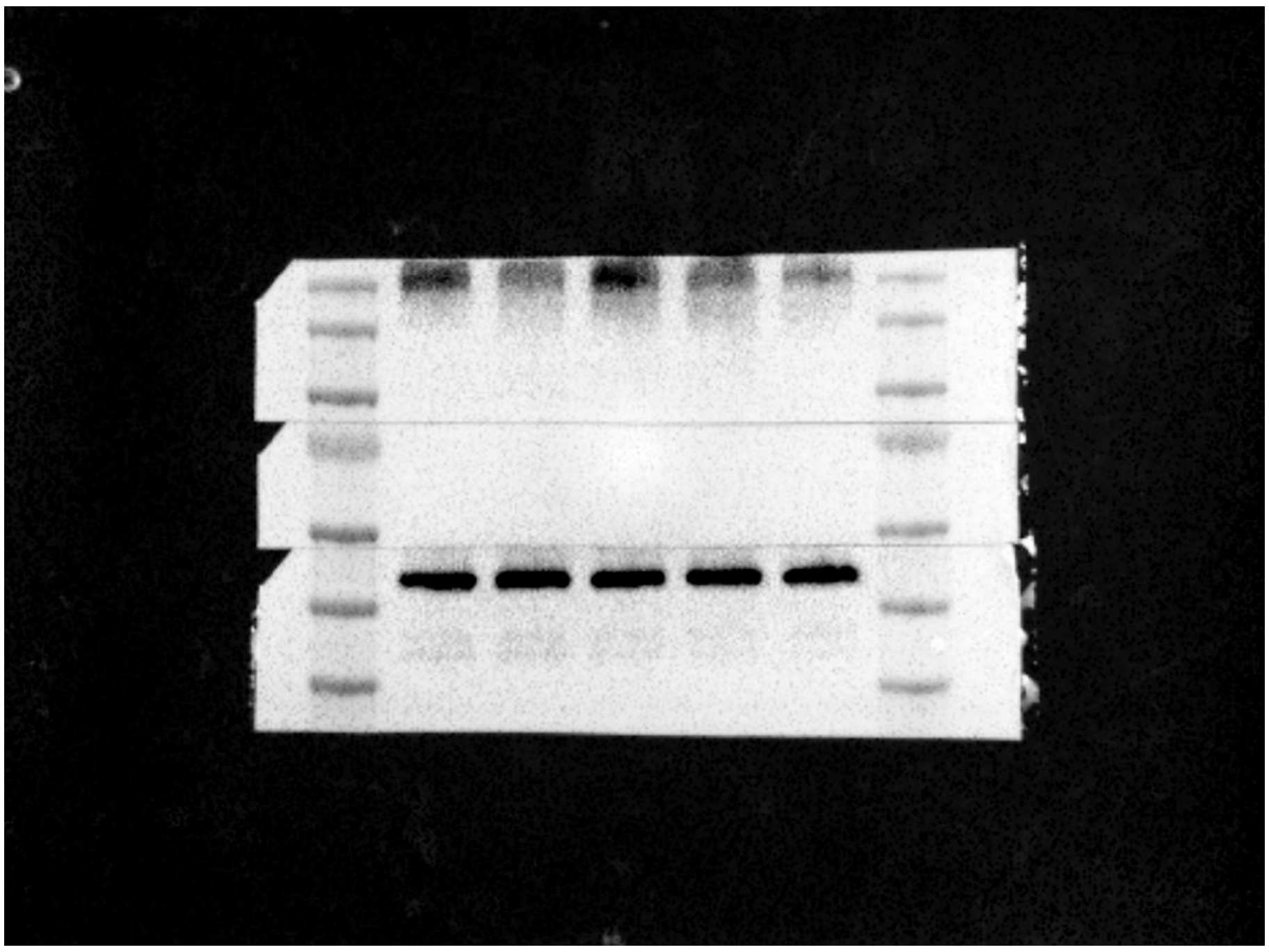

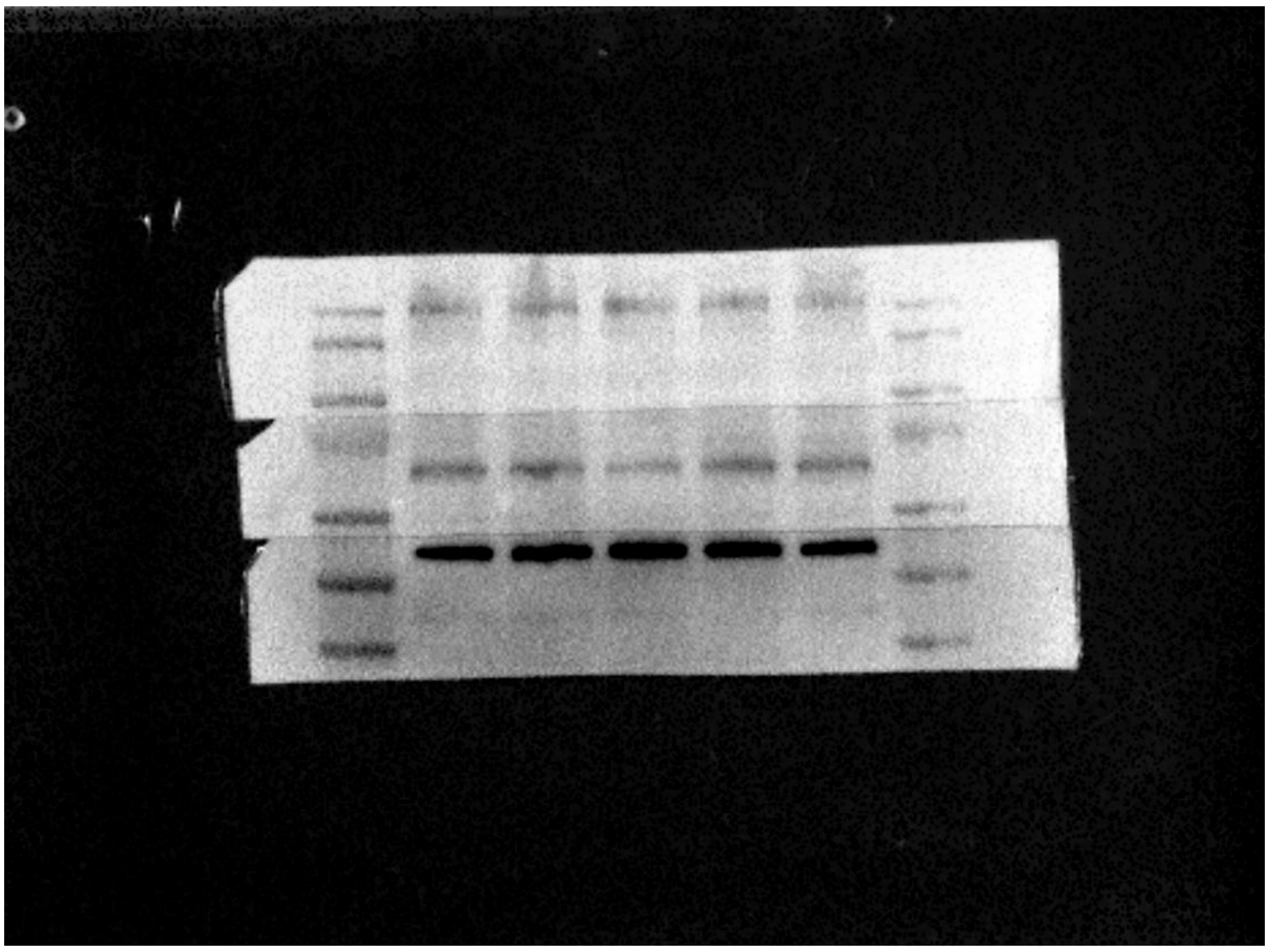

Supplement: Supplementary file 1 [file pone.0359087.s006.pdf]
